# Supplementary material for: A Wnt-mediated transformation of the bone marrow stromal cell identity orchestrates skeletal regeneration
Source: Nat Commun. 2020 Jan 16;11:332. doi: 10.1038/s41467-019-14029-w (PMC6965122; doi:10.1038/s41467-019-14029-w)
Supplement: Supplementary file 1 — Supplementary Information [file 41467_2019_14029_MOESM1_ESM.pdf]

Supplementary Information

A Wnt-mediated transformation of the bone marrow stromal cell identity orchestrates skeletal regeneration

Matsushita et al.

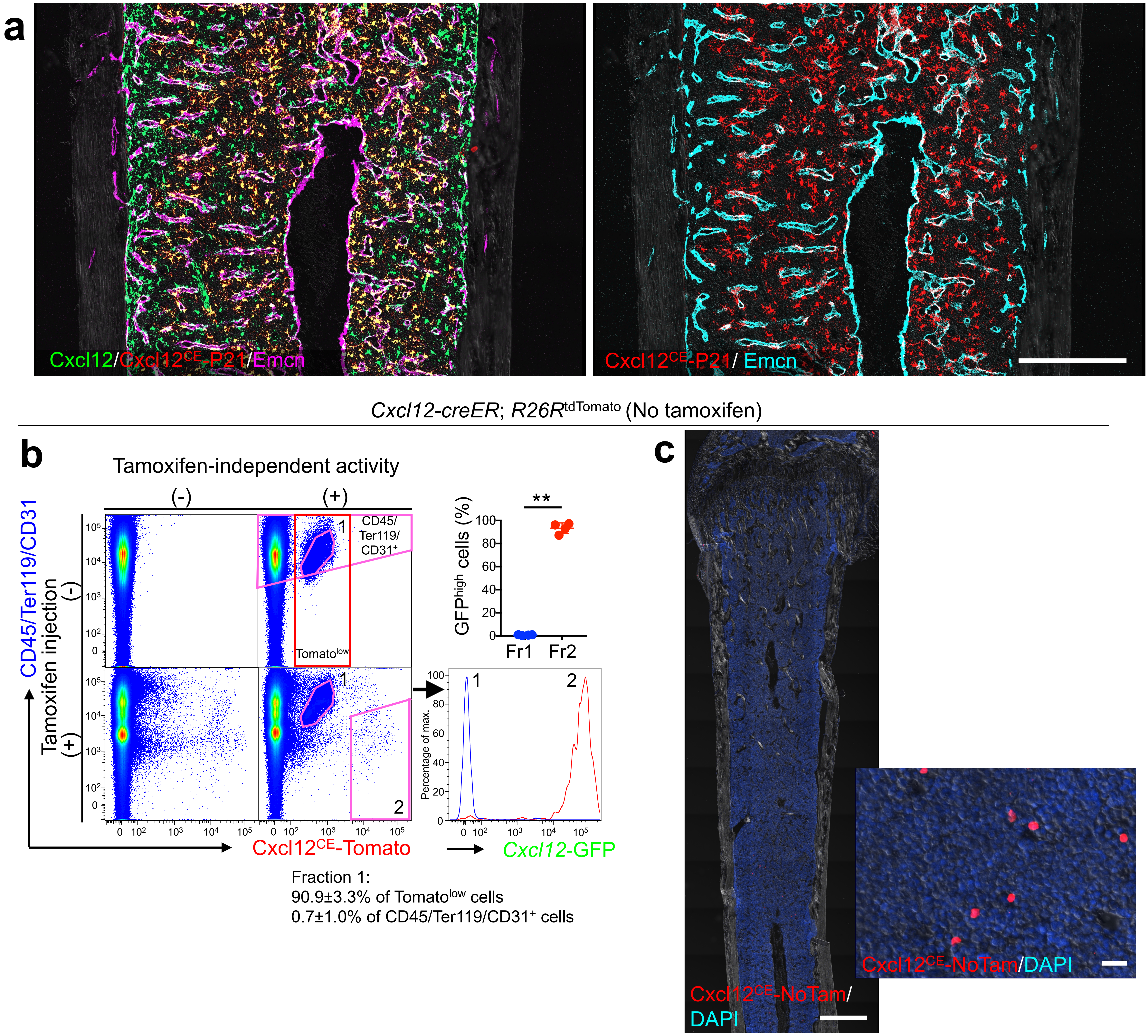

Supplementary Figure 1. Characterization of *Cxcl12-creER* line

(a) Short-chase analysis of *Cxcl12-creER*$^{+}$ cells. *Cxcl12*$^{GFP/+}$*; Cxcl12-creER*; *R26R*^{tdTomato} (pulsed at P21) distal femurs. Emcn staining. Grey: DIC. Scale bar: 500μm. *n*=5 mice.
(b,c) Tamoxifen-negative control of *Cxcl12-creER*; *R26R*^{tdTomato} mice at P28. (b): Flow cytometry analysis of FSC/SSC-gated bone marrow cells. Left panels: representative plots of cells from mice without tamoxifen-independent recombination, right panels: those from mice with some tamoxifen-independent activities. Upper panels: without tamoxifen, lower panels: with tamoxifen injection at P21. Fraction 1: CD45/Ter119/CD31^{+}tdTomato^{low} tamoxifen-independently induced hematopoietic population. Fraction 2: CD45/Ter119/CD31^{neg}tdTomato^{high} tamoxifen-induced mesenchymal population. Upper right panel: percentage of Cxcl12-GFP^{high} cells among Fraction 1 and Fraction 2. *n*=4 mice. $^{**}p<0.01$, two-tailed, Mann-Whitney's *U*-test. Data are presented as mean ± s.d. (c): Distal femur bone marrow with growth plates on top. Right panel: central marrow space. Gray: DIC. Scale bar: 500μm (left), 20μm (right). *n*=4 mice. Source data are provided as a Source Data file.

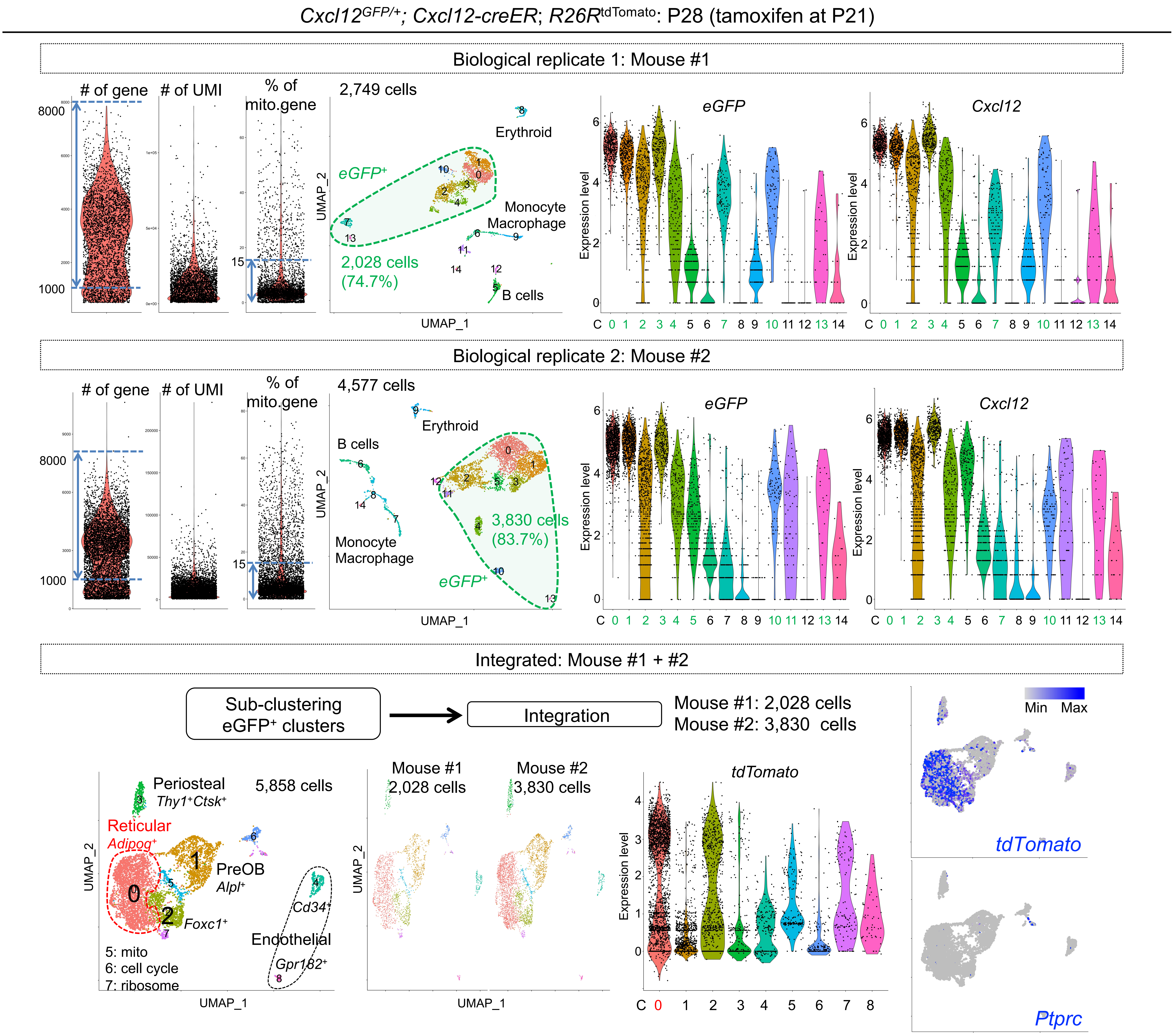

Supplementary Figure 2. Single cell RNA-seq analysis of Cxcl12-creER⁺ BMSCs

Single cell RNA-seq analysis of fluorescently sorted single cells gated on a GFP^{high} fraction harvested from *Cxcl12* $^{GFP/+}$; *Cxcl12-creER*; *R26R*^{tdTomato} femur bone marrow at P28 (pulsed at P21). Upper panels: Biological replicate 1 (Mouse #1, *n*=2,749 cells), middle panels: Biological replicate 2 (Mouse #2, *n*=4,577 cells), bottom panels: integrated data of the two biological replicates. Leftmost panels: QC plots, left center panels: UMAP-based visualization of major classes of FACS-sorted cells, right center panels: violin plots, right panels: feature plots. Green dotted contours: *eGFP*⁺ clusters. Lower right center panel: *tdTomato* expression in each cluster in the integrated space. *n*=5,858 cells merged from two biological replicates (Mouse #1: 2,028 cells, Mouse #2: 3,830 cells).

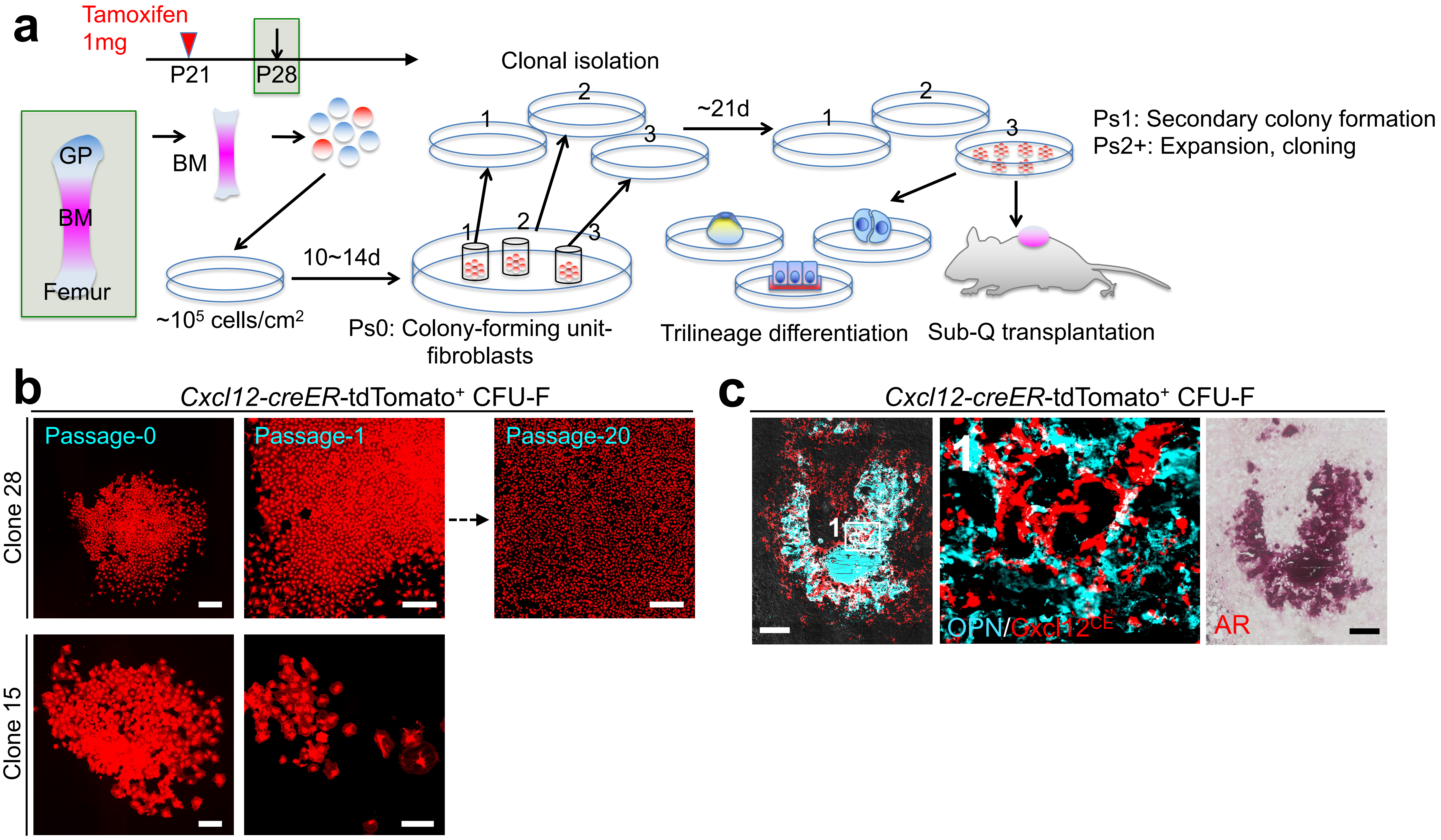

Supplementary Figure 3. ***Cxcl12-creER*** **marks a small subset of CFU-Fs**

(a) *In vitro* assays on self-renewal and differentiation of individual *Cxcl12-creER*$^{+}$ CFU-Fs. Bone marrow cells were isolated from *Cxcl12-creER*; *R26R*^{tdTomato} mice at P28 (pulsed at P21), and cultured at a clonal density (Ps0: Passage 0). Primary colonies were isolated individually and further cultured independently (Ps1: Passage 1). Secondary colonies were passaged further (Ps2: Passage 2 and higher) and cultured under trilineage differentiation conditions *in vitro* or subcutaneously transplanted into immunodeficient mice.
(b) Self-renewing (Clone 28) and non-self-renewing (Clone 15) Cxcl12^{CE}-tdTomato^{+} clones. Scale bar: 200µm.
(c) Transplantation assay of Cxcl12^{CE}-tdTomato^{+} clones into immunodeficient mice, 8 weeks after transplantation. (1): boxed area of leftmost panel. OPN: osteopontin, Light blue: OPN, red: tdTomato. Scale bar: 500µm. *n*=3 mice.

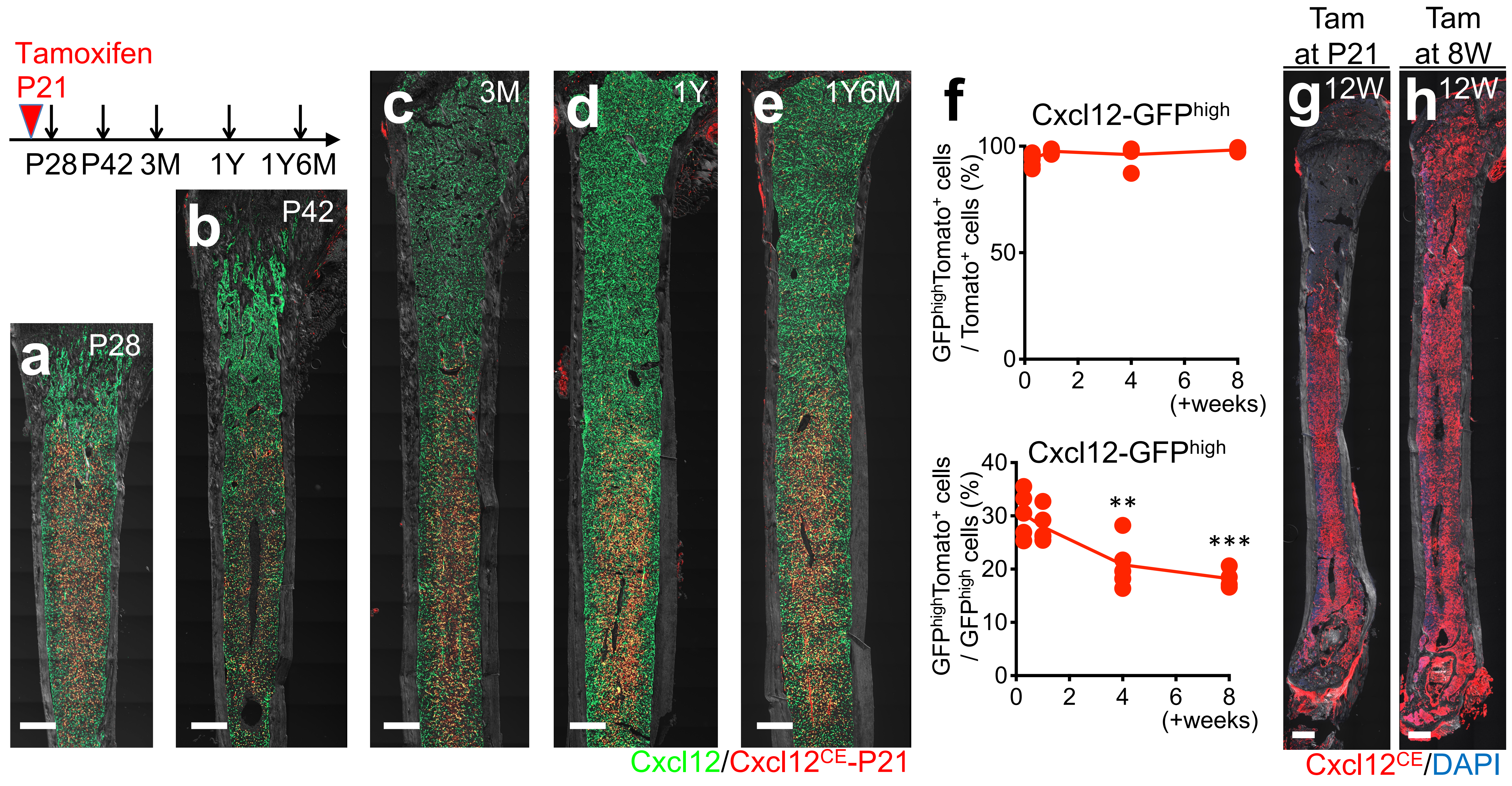

Supplementary Figure 4. Cell-fate analysis reveals dormancy of Cxcl12-creER⁺ BMSCs

(a-f) Long-chase analysis of *Cxcl12-creER*$^{+}$ reticular cells pulsed at P21. *Cxcl12*$^{GFP/+}$; *Cxcl12-creER*; *R26R*^{tdTomato} distal femurs with growth plates on top. (a-e): P21-pulsed femurs analyzed at P28 (a), P42 (b), 3M (c), 1Y (d) and 1Y6M (e). Grey: DIC. Scale bar: 500µm. *n*=3 mice per group. (f): Percentage of Cxcl12-GFP^{high}tdTomato^{+} cells per total tdTomato^{+} (upper panel) or Cxcl12-GFP^{high} BMSCs (lower panel) by using flow cytometry. *n*=6 (+2 days), *n*=5 (+1W, +4W) and *n*=4 (+8W) mice per group. ***p<0.001, **p<0.01, +2 days versus +4W, mean difference = 9.513, 95% confidence interval (3.355, 15.67); +2 days versus +8W, mean difference = 12.13, 95% confidence interval (5.568, 18.70). Two tailed, One-way ANOVA followed by Tukey's post-hoc test. Data are presented as mean ± s.d. Source data are provided as a Source Data file.
(g,h) Whole bone images of *Cxcl12-creER; R26R*^{tdTomato} femurs at 12W, pulsed at P21 (g) and at 8W (h). Grey: DIC. Scale bar: 500µm.

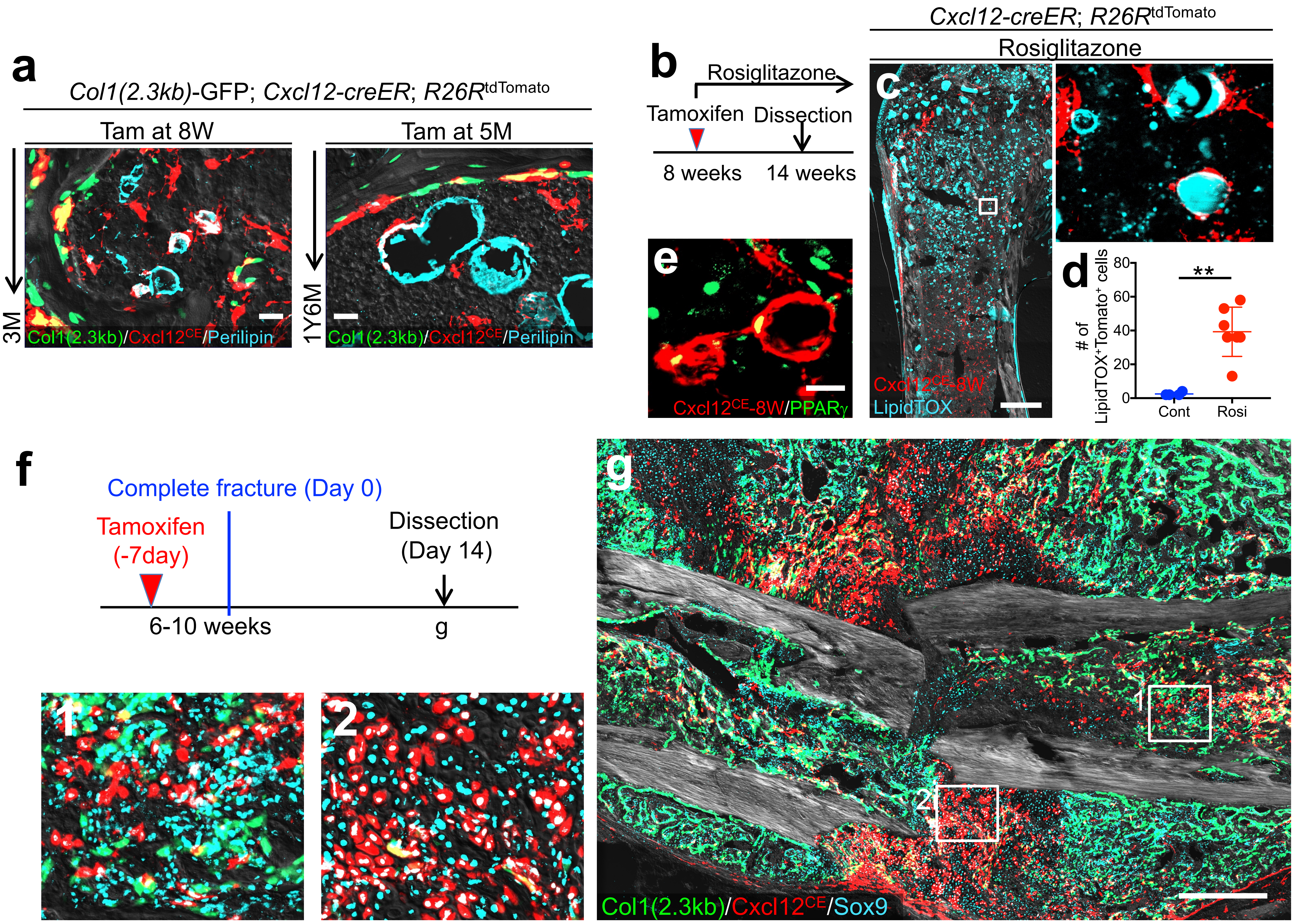

Supplementary Figure 5. Cxcl12-creER⁺ BMSCs in adipogenesis and fracture healing

(a) Spontaneous marrow adipogenesis at 3M and 1Y6M. *Col1(2.3kb)*-GFP; *Cxcl12-creER*; *R26R*^{tdTomato} femurs (pulsed at 8W or 5M). Grey: DIC. Scale bar: 20µm. *n*=3 mice.
(b-e) Inductive marrow adipogenesis using PPARγ agonist rosiglitazone (Rosi). (b): *Cxcl12-creER*; *R26R*^{tdTomato} femurs (pulsed at 8W) treated with Rosi for 6 weeks. (c): LipidTOX staining. Grey: DIC. Scale bar: 500µm. (d): Quantification of LipidTOX^{+}tdTomato^{+} cells, *Cxcl12-creER*; *R26R*^{tdTomato} mice (pulsed at 8W) with control (Cont) or Rosi diet. *n*=4 (Cont), *n*=7 (Rosi) mice. ***p*<0.01, two-tailed, Mann-Whitney's *U*-test. Data are presented as mean ± s.d. Source data are provided as a Source Data file. (e): PPARγ staining. Scale bar: 20µm.
(f,g) Tibial complete fracture experiments. *Col1(2.3kb)*-GFP*; Cxcl12-creER; R26R*^{tdTomato} mice received a tamoxifen pulse at 6 – 10 weeks of age, underwent surgery at 7 days after tamoxifen injection. Fourteen days after surgery. Grey: DIC. Scale bar: 500µm. *n*=4 mice.

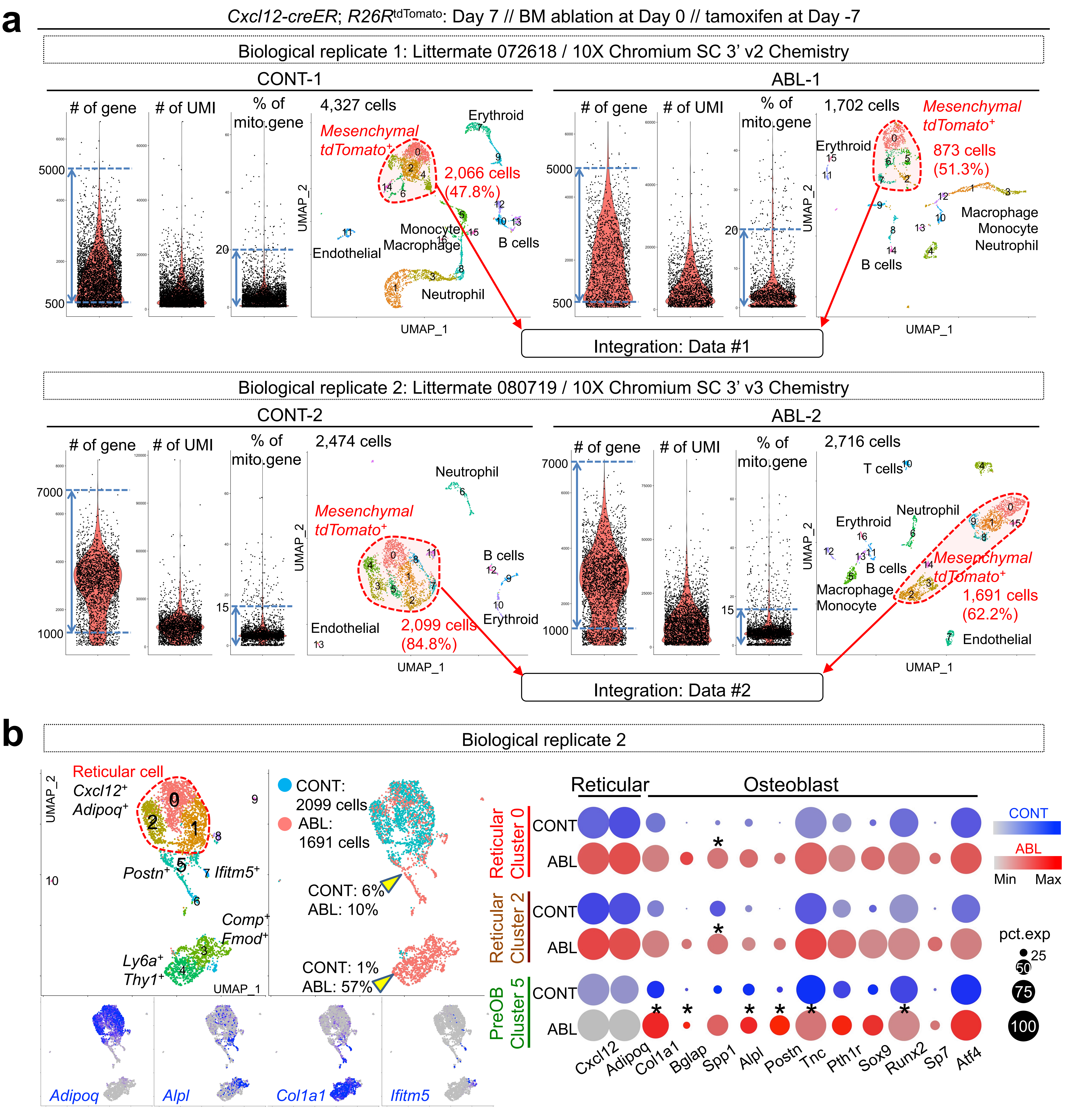

Supplementary Figure 6. Injury-induced identity conversion of Cxcl12-creER+ BMSCs

(a) Single cell RNA-seq analysis of fluorescently sorted single cells gated on a tdTomato^{high} fraction harvested from *Cxcl12-creER*; *R26R*^{tdTomato} contralateral (CONT) or ablated (ABL) femur bone marrow after 7 days of surgery (pulsed at 7 days before surgery). Upper panels: Biological replicate 1 (Littermate 072618, CONT-1: 4,327 cells, ABL-1: 1,702 cells), lower panels: Biological replicate 2 (Litter mate 080719, CONT-2: 2,474 cells, ABL-2: 2,716 cells). Left two panels: CONT cells (CONT-1, CONT-2), right two panels: ABL cells (ABL-1, ABL-2). Each left panels: QC plots, each right panels: UMAP-based visualization of major classes of FACS-sorted cells. Red dotted contours: mesenchymal tdTomato^{+} clusters. Each biological replicate pooled from *n*=7 mice.
(b) UMAP-based visualization of major classes of mesenchymal Cxcl12^{CE}-tdTomato^{+} cells (Cluster 0 – 10), Biological replicate 2. Two datasets were integrated by Seurat/CCA. Leftmost upper panel, red dotted contour: reticular (Cluster 0–2). Left lower panels: feature plots. Blue: high expression. Left center upper panel: cells colored by conditions (CONT, ABL). Biological replicate 2, *n*=3,790 cells (CONT: 2,099 cells, ABL: 1,691 cells), pooled from *n*=7 mice. (E): Split-dot-based visualization of representative gene expression. Cluster 0,2 (reticular) and Cluster 5 (pre-osteoblast) shown for reticular-signature genes (*Cxcl12*, *Adipoq*) and osteoblast-signature genes (*Col1a1, Bglap, Spp1, Alpl, Postn, Tnc, Pth1r, Sox9, Runx2, Sp7, Atf4*). *p<0.0001, Wilcoxon rank sum test. Circle size: percentage of cells expressing a given gene in a given cluster (0 – 100%), Color density: expression level of a given gene.

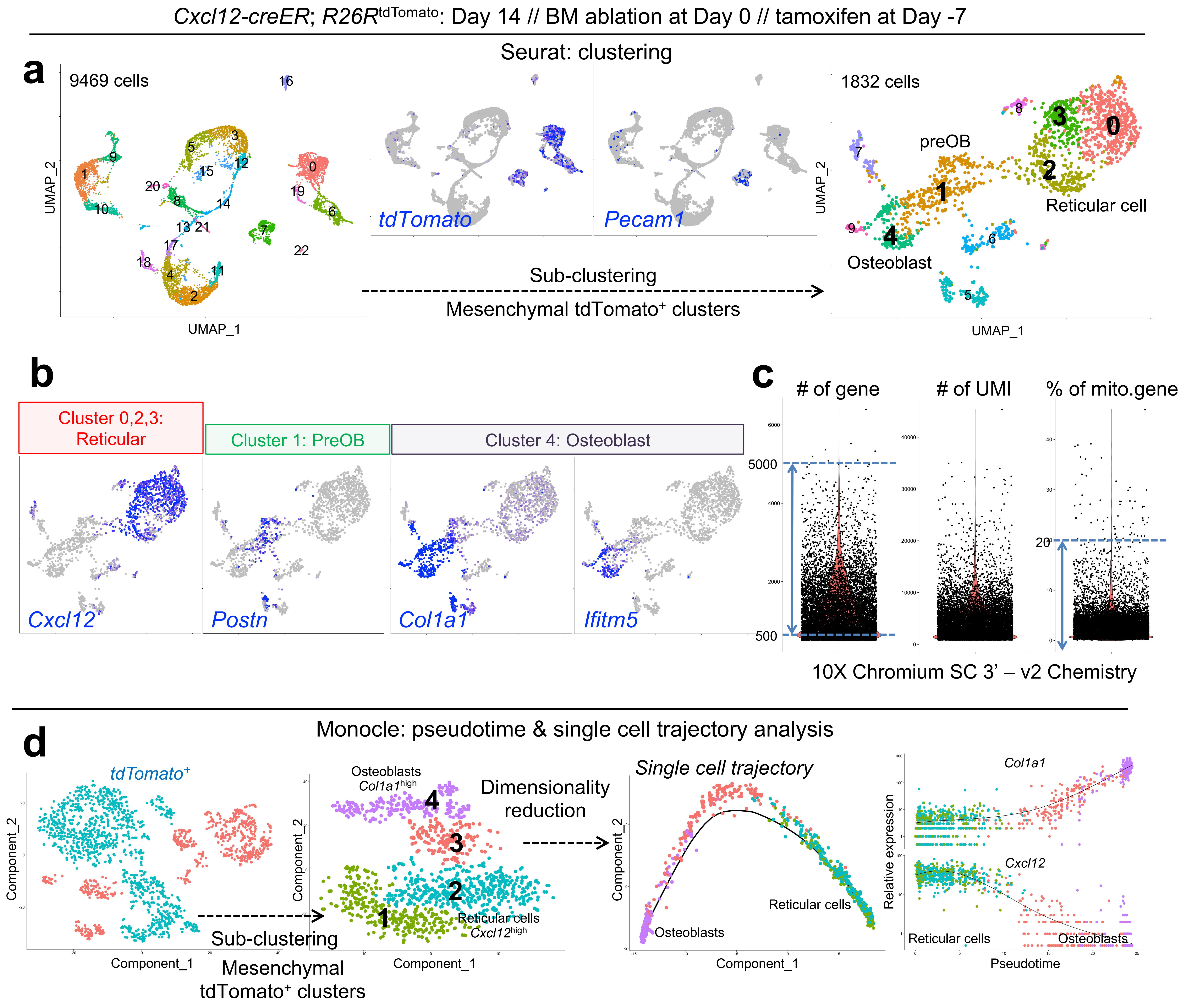

Supplementary Figure 7. A single cell trajectory of Cxcl12-creER^{+} BMSCs in regeneration

(a,b) Clustering analyses by Seurat. (a): UMAP-based visualization of major classes of FACS-sorted cells (left) and mesenchymal Cxcl12^{CE}-tdTomato^{+} cells (right) (Cluster 0 – 9). Cluster 0,2,3: reticular cells, Cluster 1: osteoblast precursors (preOB), Cluster 4: osteoblast. Feature plots: *tdTomato* (left) and *Pecam1* (right). (b): Feature plots of genes enriched in each cluster. *n*=1,832 cells pooled from *n*=7 mice.
(c) Standard QC plots. Number of genes (left) and UMIs (center) detected, percentage of mitochondrial genes (right).
(d) Pseudotime and single cell trajectory analysis by Monocle. Left panels: sub-clustering of mesenchymal tdTomato^{+} cells. To isolate mesenchymal tdTomato^{+} cells by excluding contaminating hematopoietic cells, two purification steps were performed: 1. Both *Col1a1*$^{+}$ and *Cxcl12*$^{+}$ clusters were extracted from the original single cell dataset. 2: Only tdTomato^{+} clusters were further extracted. The remaining mesenchymal tdTomato^{+} clusters were further sub-clustered (Cluster 1-4) for single cell trajectory analysis. Center panel: single cell trajectories after dimensionality reduction. Right panel: pseudotime gene plots. Cluster 1,2: Reticular cells (*Cxcl12* > 30), Cluster 3: intermediate state (*Cxcl12* < 30, *Col1a1* < 200), Cluster 4: Osteoblasts (*Col1a1* > 200).

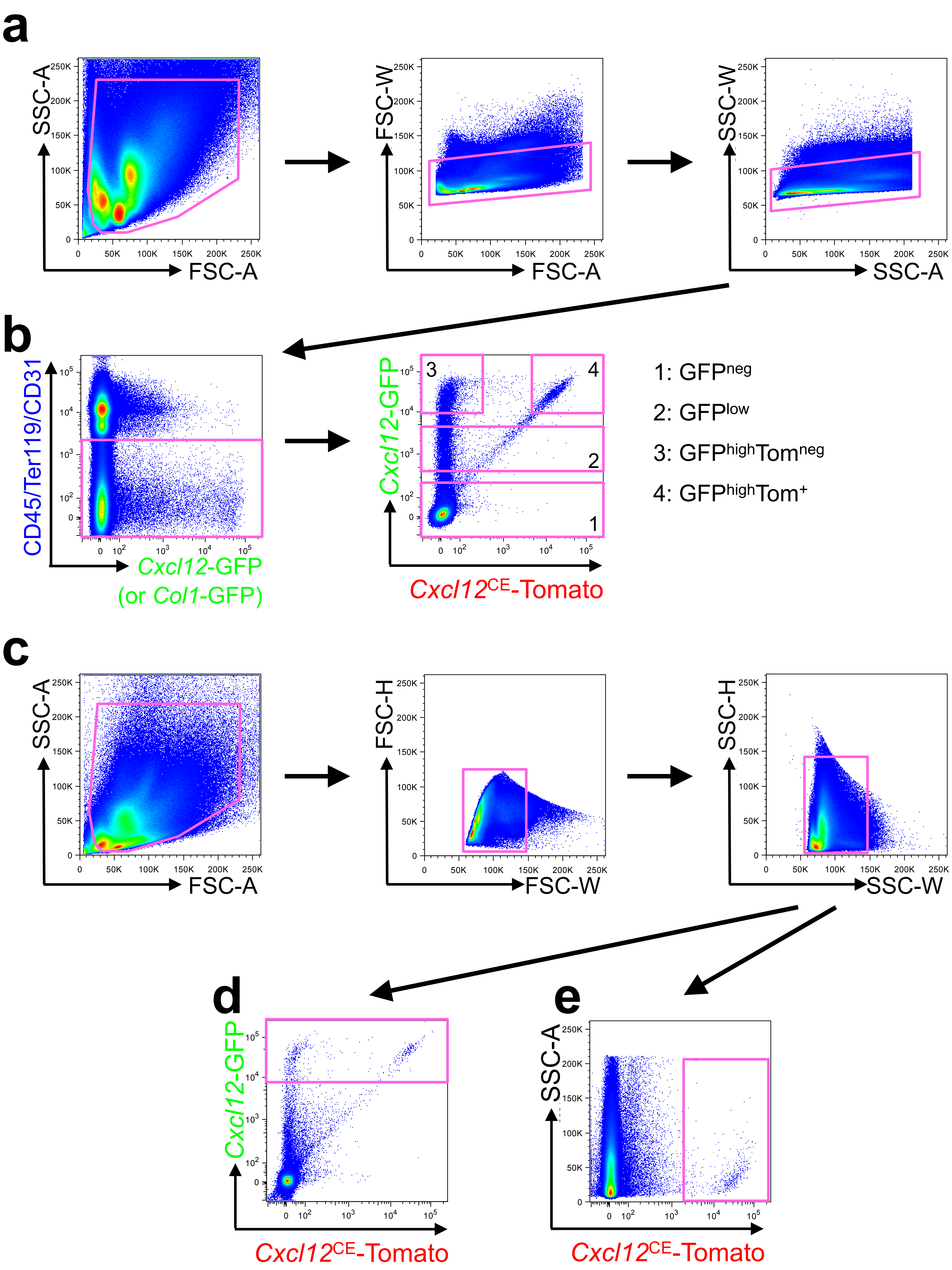

Supplementary Figure 8. Gating strategies for flow cytometry analysis and cell sorting

(a,b) Flow cytometry analysis of CD45/Ter119/CD31^{neg} cells at various time points. Gating strategy for BMSCs and osteoblasts/cytes isolated from *Cxcl12*$^{GFP/+}$ or *Col1(2.3kb)*-GFP; *Cxcl12-creER*; *R26R*^{tdTomato} femurs.
(**Fig.1f,g, j-m,o, Fig.3h,k, Fig.5i, Fig.7a, Supplementary Fig.1b, 4f**)

(c-e) Cell sorting strategy of Cxcl12-GFP^{+}Cxcl12-creER^{+} cells isolated from *Cxcl12*$^{GFP/+}$; *Cxcl12-creER*; *R26R*^{tdTomato} femurs (c,d) or Cxcl12-creER^{+} cells isolated from *Cxcl12-creER*; *R26R*^{tdTomato} femurs for single cell RNA-seq analysis (c,e).
(**Fig.2a, Fig.6a, Fig.7d**)
