# Supplementary material for: A Wnt-mediated transformation of the bone marrow stromal cell identity orchestrates skeletal regeneration
Source: Nat Commun. 2020 Jan 16;11:332. doi: 10.1038/s41467-019-14029-w (PMC6965122; doi:10.1038/s41467-019-14029-w)
Supplement: Supplementary file 3 — Reporting Summary [file 41467_2019_14029_MOESM3_ESM.pdf]

nature research

Corresponding author(s): Noriaki Ono

Last updated by author(s): Nov 1, 2019

Reporting Summary

Nature Research wishes to improve the reproducibility of the work that we publish. This form provides structure for consistency and transparency in reporting. For further information on Nature Research policies, see Authors & Referees and the Editorial Policy Checklist.

Statistics

For all statistical analyses, confirm that the following items are present in the figure legend, table legend, main text, or Methods section.

n/a	Confirmed	
☐	☒	The exact sample size (*n*) for each experimental group/condition, given as a discrete number and unit of measurement
☐	☒	A statement on whether measurements were taken from distinct samples or whether the same sample was measured repeatedly
☐	☒	The statistical test(s) used AND whether they are one- or two-sided<br>*Only common tests should be described solely by name; describe more complex techniques in the Methods section.*
☐	☒	A description of all covariates tested
☐	☒	A description of any assumptions or corrections, such as tests of normality and adjustment for multiple comparisons
☐	☒	A full description of the statistical parameters including central tendency (e.g. means) or other basic estimates (e.g. regression coefficient) AND variation (e.g. standard deviation) or associated estimates of uncertainty (e.g. confidence intervals)
☐	☒	For null hypothesis testing, the test statistic (e.g. *F*, *t*, *r*) with confidence intervals, effect sizes, degrees of freedom and *P* value noted<br>*Give P values as exact values whenever suitable.*
☒	☐	For Bayesian analysis, information on the choice of priors and Markov chain Monte Carlo settings
☒	☐	For hierarchical and complex designs, identification of the appropriate level for tests and full reporting of outcomes
☒	☐	Estimates of effect sizes (e.g. Cohen's *d*, Pearson's *r*), indicating how they were calculated

Our web collection on statistics for biologists contains articles on many of the points above.

Software and code

Policy information about availability of computer code

Data collection	ZEN 2 (blue edition, ZEISS), FACSDiva (BD), Cell Ranger 2.2 (10X Genomics)
Data analysis	ZEN 2 (blue edition, ZEISS), Image J (NIH), FlowJo 9.3.3 (TreeStar), GraphPad Prism 7.0, R 3.4, Seurat 2.3.4, Monocle 2.8.0

For manuscripts utilizing custom algorithms or software that are central to the research but not yet described in published literature, software must be made available to editors/reviewers. We strongly encourage code deposition in a community repository (e.g. GitHub). See the Nature Research guidelines for submitting code & software for further information.

Data

Policy information about availability of data

All manuscripts must include a data availability statement. This statement should provide the following information, where applicable:

- Accession codes, unique identifiers, or web links for publicly available datasets
- A list of figures that have associated raw data
- A description of any restrictions on data availability

The datasets generated during and/or analyzed during the current study are available from the corresponding author on reasonable request. The single cell RNA-seq datasets described in this publication have been deposited in the NCBI's Gene Expression Omnibus

Field-specific reporting

Please select the one below that is the best fit for your research. If you are not sure, read the appropriate sections before making your selection.

☒ Life sciences ☐ Behavioural & social sciences ☐ Ecological, evolutionary & environmental sciences

Life sciences study design

All studies must disclose on these points even when the disclosure is negative.

Sample size	No statistical method was used to predetermine sample size. We chose the numbers of mice to study based on our prior experience that give good standard errors of the mean and good statistics to make it unlikely that we miss a biologically important difference between groups.
Data exclusions	Some of the data were excluded from the study because of the pre-established criteria such as problems or failures in identifying correct genotypes or birth dates, and issues unrelated to the intervention of the study such as spontaneous malnutrition. In any case, we consistently used littermate controls with corresponding genotypes in analysis.
Replication	For all data presented in the manuscript, we examined at least three independent biological samples (three different mice) to ensure the reproducibility. For each series of the experiments, all attempts at replication were successful.
Randomization	The experiments were not randomized. We used all the available mice of the desired genotypes. Mice were allocated to particular groups based on results of PCR-genotyping typically performed around one week after birth. Covariates were controlled by considering multiple factors, such as genotypes and general phenotypical data (i.e. body weight). On principle, we did not observe any particular difference among groups.
Blinding	The investigators were not blinded to allocation during experiments and outcome assessment because it was impossible due to following reasons: samples were allocated to particular groups before experiments were initiated based on genotyping results, and given unique identifiers highlighting groups throughout experiments i.e. housing in cages, tissue collections, sample preparation and data acquisition. However, we did not pay particular attention to groups when we were measuring and counting.

Reporting for specific materials, systems and methods

We require information from authors about some types of materials, experimental systems and methods used in many studies. Here, indicate whether each material, system or method listed is relevant to your study. If you are not sure if a list item applies to your research, read the appropriate section before selecting a response.

Materials & experimental systems

n/a	Involved in the study	
☐	☒	Antibodies
☒	☐	Eukaryotic cell lines
☒	☐	Palaeontology
☐	☒	Animals and other organisms
☒	☐	Human research participants
☒	☐	Clinical data

Methods

n/a	Involved in the study	
☒	☐	ChIP-seq
☐	☒	Flow cytometry
☒	☐	MRI-based neuroimaging

Antibodies

Antibodies used

ThermoFisher/eBioscience
eFlour450-conjugated CD31 (390, Cat# 48-0311-82, Lot# 4301770)
eFlour450-conjugated CD45 (30F-11, Cat# 48-0451-82, Lot # 4295770)
eFlour450-conjugated Ter119 (TER-119, Cat# 48-5921-82, Lot# 4295840)
Allophycocyanin (APC)-conjugated CD31 (390, Cat# 17-0311-82, Lot# 4330203)
Allophycocyanin (APC)-conjugated CD45 (30F-11, Cat# 17-0451-82, Lot # 1966484)
Allophycocyanin (APC)-conjugated Ter119 (TER-119, Cat# 17-5921-82, Lot# 4295921)
Allophycocyanin (APC)-conjugated CXCL12 (Cat# IC350A, Lot# ABFS0215041)

ThermoFisher/Invitrogen
Rabbit anti PPAR gamma monoclonal antibody (K.242.9, Cat#MA5-14889, Lot# SA2329903)
Alexa Fluor 647 donkey anti-rabbit IgG (H+L) (Cat# A31573, Lot# 1322326)
Alexa Fluor 633 goat anti-rat IgG (H+L) (Cat# A21049, Lot# 679064)
Alexa Fluor 647 donkey anti-goat IgG (H+L) (Cat# A21082, Lot# 1301819)

EMD-Millipore
Rabbit anti-Sox9 polyclonal antibody (Cat# AB5535, Lot# 2922429)

R & D systems
Goat anti LepR polyclonal antibody (Cat# AF497, Lot# BFU0216101)
Goat anti Osteopontin polyclonal antibody (Cat# AF808, Lot# BDO0617041)
Goat anti ALPL polyclonal antibody (Cat# AF2910, Lot# WYM0116081)

Sigma

	Rabbit anti perilipinA/B polyclonal antibody (Cat#1873, Lot# 018M4869V)<br><br>Bio-Rad<br>Rat anti CD31 antibody (ER-MP12, Cat# MCA2388, Lot# 1608)<br><br>Santa Cruz Biotechnology<br>Rat anti-endomucin (Emcn) monoclonal antibody (Cat# sc65495, Lot# C2816)<br><br>Bioss<br>Rabbit ant SCF polyclonal antibody (Cat# bs-0545R, Lot# AC030201)
Validation	More detailed information about these antibodies is available on these manufacturers' websites.

Animals and other organisms

Policy information about studies involving animals; ARRIVE guidelines recommended for reporting animal research

Laboratory animals	We used genetically modified mice (mus musculus) for this study. Most of the mouse line have been backcrossed to C57BL/6 background. We used female breeder mice in a FVB/N background. Mice with both sexes were used throughout their lifespan (up to 2 years of age). Mouse strains used in the study were as following: Cxcl12-creER, Osx-creER, Cxcl12- GFP/null, Rosa26-CAG-loxP-stop-loxP-tdTomato (Ai14: R26R-tdTomato, JAX007914), Rosa26-SA-loxP-GFP-stop-loxP-DTA (JAX006331), Ubc-creER (JAX007001), Dlx5-creER (JAX010705), Col1a1(2.3kb)-GFP (JAX013134), Osteocalcin(3.8kb)-GFPtpz (JAX017469), Ctnnb-floxed (JAX004152), Apc-floxed (JAX009045), Runx2-floxed, Sox9-floxed (JAX013106)
Wild animals	N/A
Field-collected samples	N/A
Ethics oversight	All procedures were conducted in compliance with the Guidelines for the Care and Use of Laboratory Animals approved by the University of Michigan's Institutional Animal Care and Use Committee (IACUC), protocol 7681 (Ono) and 8779 (Kozloff).

Note that full information on the approval of the study protocol must also be provided in the manuscript.

Flow Cytometry

Plots

Confirm that:

- [x] The axis labels state the marker and fluorochrome used (e.g. CD4-FITC).
- [x] The axis scales are clearly visible. Include numbers along axes only for bottom left plot of group (a 'group' is an analysis of identical markers).
- [x] All plots are contour plots with outliers or pseudocolor plots.
- [x] A numerical value for number of cells or percentage (with statistics) is provided.

Methodology

Sample preparation	Soft tissues and epiphyses were carefully removed from dissected femurs. After removing distal epiphyseal growth plates and cutting off proximal ends, femurs were cut roughly and incubated with 2 Wunsch units of Liberase TM and 1mg of Pronase (Sigma/Roche 10165921001) in 2ml Ca2+, Mg2+-free HBSS at 37oC for 60 min on a shaking incubator (ThermomixerR, Eppendorf). After cell dissociation, cells were mechanically triturated using an 18-gauge needle with a 1ml Luer-Lok syringe (BD) and a pestle with a mortar (Coors Tek), and subsequently filtered through a 70µm cell strainer (BD) into a 50ml tube on ice to prepare single cell suspension. These steps were repeated for 5 times, and dissociated cells were collected in the same tube. Cells were pelleted and resuspended in an appropriate medium for subsequent purposes.
Instrument	BD LSR Fortessa (BDBiosciences)
Software	FACSDiva v8.0.1 (BD) & FlowJo 9.3.3 (TreeStar) software
Cell population abundance	N/A
Gating strategy	Single cells were first gated using FSC and SSC denominators. Negative 'unstained' control samples were always used as a reference to determine the demarcation between the positive and negative populations.

- [x] Tick this box to confirm that a figure exemplifying the gating strategy is provided in the Supplementary Information.
